# Knowledge, risk-perception, and uptake of COVID-19 prevention measures in sub-Saharan Africa: a scoping review

**DOI:** 10.4314/ahs.v22i3.59

**Published:** 2022-09

**Authors:** Joseph KB Matovu, Alex Mulyowa, Rogers Akorimo, Daniel Kirumira

**Affiliations:** 1 Department of Disease Control and Environmental Health, School of Public Health, College of Health Sciences, Makerere University, Kampala, Uganda; 2 Department of Community and Public Health, Busitema University Faculty of Health Sciences, Mbale, Uganda

**Keywords:** COVID-19, prevention, sub-Saharan Africa

## Abstract

**Background:**

The COVID-19 pandemic has almost affected the entire globe and is currently in a resurgent phase within the sub-Saharan African region.

**Objective:**

This paper presents results from a scoping review of literature on knowledge, risk-perception, conspiracy theories and uptake of COVID-19 prevention measures in sub-Saharan Africa.

**Methods:**

We used the following search terms: ‘COVID-19’, ‘knowledge’, ‘perceptions’, ‘perspectives’, ‘misconceptions’, ‘conspiracy theories’, ‘practices’ and ‘sub-Saharan Africa’. Basing on the Preferred Reporting Items for Systematic Reviews and Meta-Analyses extension for scoping reviews (PRISMA-ScR) guidelines, we identified 466 articles for review; 36 articles met the inclusion criteria. We extracted data on knowledge, risk-perception, conspiracy theories and uptake of COVID-19 primary prevention measures.

**Results:**

Knowledge of COVID-19 was high (91.3–100%) and associated with age and education; risk-perception was equally high (73.3–86.9%) but varied across studies. Uptake of hand-washing with water and soap or hand-sanitizing ranged between 63–96.4%, but wearing of face masks and social distancing fared poorly (face masks: 2.7%–37%; social distancing: 19–43%).

**Conclusion:**

While knowledge of COVID-19 is nearly universal, uptake of COVID-19 prevention measures remains sub-optimal to defeat the pandemic. These findings suggest a need for continued health promotion to increase uptake of the recommended COVID-19 prevention measures in sub-Saharan Africa.

## Introduction

The corona virus disease (COVID-19) is a respiratory illness caused by the SARS-Cov-2 virus^1^, a new virus that has not been previously identified in humans^2^, ^3^. This virus has signs and symptoms similar to the common cold including but not limited to, fever, dry cough, shortness of breath, and chest pain. However, there have also been many asymptomatic cases of COVID-19 that have been reported. The infectious COVID-19 spreads through person to person contact through infected respiratory fluids and contaminated surfaces, causing respiratory distress and in the worst cases, death^4^. To date, COVID-19 has affected almost the entire globe with the exception of just a few countries^5^. More specifically, this pandemic has so far affected 222 countries and territories around the world^6^. As of December 6, 2021, global statistics showed that the total number of COVID-19 cases stood at 265,876,379 cases, and of these, 5,256,285 (1.98%) people had died of COVID-19^7^. In sub-Saharan Africa (SSA), the pandemic has reached every nation, with a total caseload standing at 8,471,592 cases with 224,073 (2.64%) deaths as of December 7, 2021^8^.

Currently, there is a lot of data on the etiology of the virus, and this has helped to inform the design of interventions to control its spread. The World Health Organization (WHO), for instance, has advised the public on three key prevention areas including hand-washing with soap and water/hand sanitizing, social distancing, and wearing a face mask^9^. In addition, the WHO has drafted the COVID-19 Strategic Preparedness and Response Plan which comprises 8 pillars that are aimed at guiding prevention efforts among countries^10^. Despite the existence of some of these interventions, as well as studies that have highlighted important lessons on strengthening the COVID-19 crisis management and policy responses across countries^11^, there is still a low risk-perception among populations as shown by evidence, especially on the African continent^12^, ^13^. Aduh and colleagues argue that when the perception of risk to epidemics is poor, then populations poorly comply to recommended public health measures^14^. Therefore, perceived risk influences health behaviour. For COVID-19, this poor risk-perception could be attributed to cultural and/or societal factors^15^ which inadvertently affect the uptake of prevention measures. While quite a number of studies have been conducted on different aspects relating to COVID-19 globally, most of these studies have been conducted outside Africa. As a result, most SSA countries are facing the corona virus pandemic crisis with inaccurate, incomplete, unreliable and untimely local data^16^. To this effect, contextual African data, though available, is still largely limited. Statistics show that mortality due to COVID-19 is increasing in SSA, although it remains one of the regions least affected by the pandemic^17^. However, the WHO estimates that the continuous spike of SARS-CoV-2 infections in SSA could position this continent as one whose population could be at risk of severe consequences due to the virus^18^, ^19^.

The strategies to combat the transmission of the virus within SSA are proving to be inefficient, at least in the meantime, indicating a disconnect between ideal responses and current realities^17^. Following pillar 2 of the WHO eight pillars for prevention of COVID-19^10^, countries have not invested a lot in risk communication and community engagement. As such, risk communication messages have probably not touched where it itches most. When the public perceives a lack of empathy, equity and consistency in pandemic response by the respective governments, people may gain distrust in ongoing approaches, but also become more fearful of the approaches proposed by the government^20^. Besides, increased beliefs in spiritual doctrines and/or healing could likely influence the uptake of prevention measures for this infection^21^,^22^, as has been the case with HIV and Ebola outbreaks^23^–^25^. With the current increasing caseloads, many SSA countries could face multiple challenges in controlling the virus, especially in light of social realities such as communal living and physical gatherings that bring people together^11^. These realities pose a challenge to many public health interventions such as social distancing, making it difficult to sustain them much for a long period of time^11^. To minimize the above-mentioned challenges, the global COVID-19 scientific community acted quickly to avail a cocktail of COVID-19 vaccines to control the spread of COVID-19. Global statistics show that up to 43% of the world's population is currently fully vaccinated but only 7.35% of the eligible population in SSA has been fully vaccinated^7^, ^8^. This low COVID-19 vaccination coverage in SSA suggests a need for increased promotion of non-pharmaceutical interventions to tame the tide of COVID-19 as efforts to improve coverage of COVID-19 vaccines in SSA gain momentum. However, there are no synthesized data at the moment to inform the scale-up of these interventions across countries and populations. The purpose of this scoping review is to provide a detailed synthesis of COVID-19 data to inform the continued implementation of non-pharmaceutical interventions necessary to control the spread of COVID-19 in SSA.

## Methods

This review followed the Arksey and O'Malley scoping study methodology^26^, which focuses on five scoping review phases, namely: i) Identifying the research questions ii) Identifying relevant studies iii) Article selection iv) Charting the data and v) Collating, summarizing and reporting the results. These phases are described in detail below.

### a) Identifying the research questions

The research questions that guided this scoping review took into consideration Arksey and O'Malley's^26^ recommendation on having a wide and inclusive approach of setting research questions to attain breadth in literature coverage. The main research questions for this scoping review were: ‘How much does the population in SSA know about COVID-19?’, ‘What are the individual risk-perceptions and conspiracy theories regarding COVID-19 among people in SSA?’, ‘What is the documented uptake of the recommended COVID-19 prevention measures in the local communities of SSA?’ and What other COVID-19 prevention measures are being adopted in SSA, even if some of them may not be recommended by any health authority?

### b) Identifying relevant articles

Published electronic articles/studies on COVID-19 knowledge, risk-perception, conspiracy theories and COVID-19 prevention practices in SSA were searched from Google Scholar and Pub Med. Other sources were websites of renown international organizations (WHO and UN), major world news organizations (CNN, BBC and Aljazeera) and article reference lists. These were all chosen based on their regional scope and variety in terms of available literature. No limits on date, language, or study design were applied during the database search. Keywords used in the search included ‘COVID-19’, ‘knowledge’, ‘perceptions’, ‘perspectives’, ‘misconceptions’, ‘conspiracy theories’, ‘practices’ and ‘sub-Saharan Africa’, which were used in combination.

### c) Article selection

#### Inclusion criteria

We included articles that: 1) were published in the English language, 2) were published with effect from March 2020, 3) reported qualitative and quantitative findings, and 4) focused on SSA. The consideration of articles with both qualitative and quantitative findings allowed for breadth in the review of the literature. We also considered articles from trusted websites (such as the WHO and UN websites) and news stories from reputable international newspapers.

#### Exclusion criteria

Exclusion of the literature applied if title and abstract of the literature were irrelevant to the research questions. Furthermore, when the same data were reported in more than one publication, only the article representing the most complete data was used. Articles on COVID-19 practices/uptake of COVID-19 prevention measures whose target population was health workers or in which majority of the respondents/participants were health workers were excluded.

#### Title and abstract relevance screening

**Figure 1:**
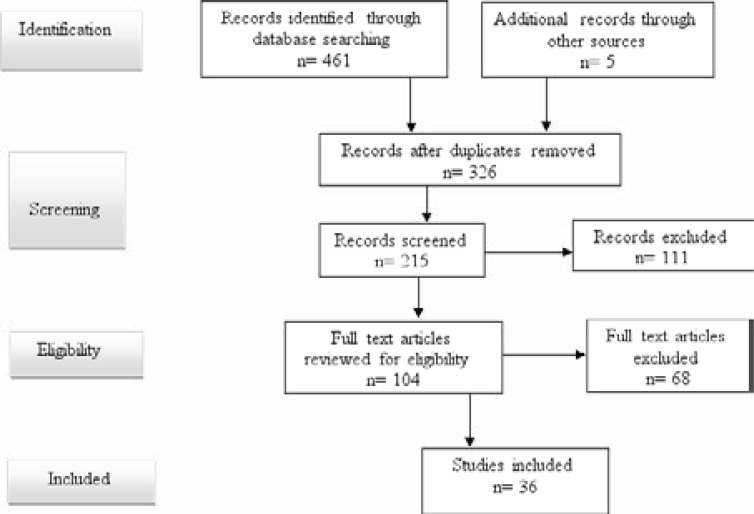
Preferred Reporting Items for Systematic Reviews and Meta-Analyses extension for scoping reviews (PRISMA-ScR) flow diagram showing article selection process

Titles and abstracts were initially screened to minimize time and resource wastage in gathering articles that did not meet the minimum inclusion criteria. Titles for which an abstract was missing were included for the subsequent review of the full-text article. As recommended by^27^, the study selection process is presented in a Preferred Reporting Items for Systematic Review and Meta-Analyses extension for scoping reviews (PRISMA-ScR) flow-chart (see Figure 1).

### d) Charting the data

We developed a data extraction tool using Microsoft Excel where characteristics of the relevant selected studies were captured^28^. These included first author name, publication year, country, study aim, study design, study population, sample size and outcome. Retrieved references were managed using Endnote. The data extraction tool was designed in a way that ensures easy data entry, interpretation, and comparison. The process of data extraction was guided by the following themes: knowledge of COVID-19, COVID-19 risk perceptions, conspiracy theories, level of trust of COVID-19 information and uptake of COVID-19 prevention measures.

### e) Collating, summarizing and reporting the results

After critically examining the articles, summaries were generated and combined for reporting purposes. The results are reported according to the selected themes. A descriptive numerical summary of the characteristics of the included studies is performed. A table was created to reflect the overall number of studies included, study designs and settings, the results reported and countries of origin of the studies (see Table 1).

**Table 1:** Characteristics of the studies reviewed

Study	Month and Year of Publication	Country	Study aim	Study design and methods	Participants	Summary of findings
32	March 2020	Kenya	To determine the current knowledge and prevailing attitudes reported by households related to COVID-19	Quantitative survey. Data were collected using a mobile phone.	Household members (2,009)	Knowledge of cough and fever as COVID-19 symptoms was high, i.e., 77% of respondents correctly identified fever and 86% identified cough. Overall, 35% of the respondents perceived they were at high risk of contracting the COVID-19 infection. Government TV adverts and short text messaging services (SMS) were the most common, trusted sources of information. Exposure to these sources was significantly higher for those with higher levels of education, at 81%.
33	June 2020	Nigeria	To investigate COVID-19 related knowledge, attitudes and practices as well as misconceptions in Katsina state.	Quantitative online survey. Data were collected us ing WhatsApp media platform.	Internet users (722)	Knowledge of the disease was high at 80%. Higher education was associated with good practices. Majority, 83%, of the respondents held at least one conspiracy theory related to COVID-19. The most frequent conspiracy theories were that 36% of the respondents believed that COVID-19 was created in a laboratory, aimed at depopulating the world (33%).
34	June 2020	Nigeria	To assess the knowledge, attitude and perception about COVID-19 among members of staff of a university community in southwest Nigeria.	Quantitative online survey	Teaching and non-teaching staff (227)	Most, 85.3%, believed that COVID-19 was a biological weapon. Knowledge level about the disease was high at 70.8%, and attitudes of the study participants were positive, at 83.1%.
35	April 2020	N/A	False information about COVID- 19 in Africa.	N/A	Article	Steaming & alcohol consumption were reported as misconceptions to cure of COVID-19; but also, some African communities believed that the disposable blue facemasks were contaminated with the virus.
36	March 2020	Ethiopia	To assess what undergraduate students at Debre Berhan knew about COVID-19 and how it shaped their attitude and practices towards the disease.	Quantitative survey. Data were collected through face-to-face interviews.	Undergraduate students (546)	Knowledge was high (73.8%) among majority of the respondents; knowledge was significantly associated with increasing age. Nearly half (42%), of the students reported that they had no concern of being infected with the virus. Prevention practices were reported to be poor, i.e., 48% were not covering their mouth and nose while coughing and sneezing, and 56% were not maintaining the recommended social distancing.
37	April 2020	Egypt	To assess knowledge, perceptions and attitudes of the Egyptian public towards the COVID-19 disease.	Quantitative Survey. Data were collected online, as well as through personal interviews.	Non-medical Egyptian adults (559)	Knowledge was mainly gained through social media (66.9%) and internet (58.3%). Risk-perception was high (86.9%).
38	April 2020	Sudan	To assess the knowledge, attitude and practice of the Sudanese people towards COVID-19	Quantitative Survey. Data were collected through Facebook and WhatsApp.	Adult Sudanese (987)	Awareness about the COVID-19 pandemic was found to be high (91.3%) among the study population, and was significantly associated with older and better educated participants. Prevention practices such as avoidance of handshaking were low at 27%. Better practices were associated with older age and female gender.
39	May 2020	Cameroon	To assess knowledge, attitudes and practices with regard to COVID- 19 in Buea municipality, Cameroon.	Quantitative survey. Data were collected through face-to-face interviews.	Adults (545)	Risk-perception was high (73.3%) among respondents. With knowledge, 21.9% had a correct knowledge of COVID-19, 43.8% had intermediate knowledge, 34.4% had poor knowledge and 11.93% had no knowledge about the disease. Respondents only knew preventive measures that affected them directly like avoiding social gathering (31.7%) and closing of schools (34.2%). Wearing of face masks was reported by 2.7% of the population.
40	October 2020	Sudan	To measure the Sudanese population's KAP in relation to COVID-19 during the pandemic	Quantitative Online survey	Sudanese general population (2,336)	Knowledge was high (84.7%). Majority, 92%, of the participants frequently washed their hands or used antiseptic.
41	July 2020	Nigeria & Egypt	To investigate the knowledge, attitudes and perceptions of Egyptians and Nigerians towards the COVID-19 pandemic.	Quantitative survey. Data were collected through emails, WhatsApp and Facebook messenger.	Adults >17 years (1,437)	Majority (61.6%) of the respondents had good knowledge of the disease. Participants aged 18–29 years were 1.4 times more likely to be knowledgeable than other age groups. Most, 96%, of the respondents practiced self-isolation and social distancing.
42	April 2020	Uganda	To assess the awareness, knowledge, attitude and practices towards measures for prevention of the spread of COVID-19 among Ugandans.	Quantitative online survey	Literate Ugandans>18yrs (1763)	Knowledge about the disease was high at 97.6%, and similarly, attitudes and prevention practices were good at 72.4% and 85.3% respectively.
12	July 2020	Uganda	To identify possible misconceptions among males and females toward COVID-19 in Uganda.	Quantitative online survey	Male and female Ugandans (161)	Females considered infection risk, severe signs, symptoms and death to be equally distributed among genders. Men on the other hand believed they were more at risk of infection. 30.2% of respondents, particularly men, felt that COVID-19 is a “white-man's disease”. Other misconceptions reported were the promotion of prayers for divine intervention against the virus, and that malaria endemic regions would be protected.
43	April 2020	Gambia	To assess perceived severity of COVID-19 among adult population in the Gambia	Quantitative survey. Data were collected through Google Docs form and WhatsApp	Adults (206)	Risk-perception among most respondents towards the virus was reported at 62.6%. 70% had limited trust in capacity of healthcare system to effectively handle the pandemic. About half (54%) of the respondents regarded death as their biggest fear towards the COVID- 19 pandemic.
20	May 2020	Zambia	To fill the knowledge gap on the role of information sources in mediating the relationship between behavioural responses to COVID- 19 and its determinant.	Quantitative online survey	Zambian citizens (182)	Information sources were found to influence risk perception towards the healthcare system in relation, and behavioral responses towards COVID-19.
44	June 2020	Cameroon	N/A	N/A	Article	African countries came up with organic cures for the virus. Madagascar for example, came up with COVID Organics (CVO), made from the Artemisia plant.
45	May 2020	Cameroon	To assess knowledge, attitudes and practices with regard to COVID- 19 in Buea Municipality, Cameroon.	Quantitative survey. Data were collected through face-to-face interviews.	Adults (545)	Risk-perception was high (73.3%) among respondents. Participants only knew preventive measures that affected them directly like avoiding social gathering (31.7%) and closing of schools (34.2%).
46	March 2020	Nigeria	To assess knowledge and perceptions about COVID-19 among the general public in Nigeria during the initial week of the pandemic lockdown in the country.	Quantitative online survey	Citizens aged 15–70 years (1,357)	Approximately half (46.94%) of the respondents believed that COVID-19 was a biological weapon. Approximately 94% of the study participants identified contact with airborne droplets via breathing, sneezing, or coughing as the most common mode of transmission; most respondents associated COVID-19 with coughing (81.13%), fever (62.79%) and shortness of breath (73.47%). 11.86% had misconceptions such as consuming gins, ginger or herbal remedies as preventive measures. Washing hands regularly and social distancing was selected by most respondents (94.25%) as a way of preventing infection
31	March 2020	Zimbabwe	To understand community and healthcare worker perspectives on COVID-19, and Zimbabwe's policy responses.	Qualitative study	CBO representatives (4) and health workers (16)	There was information overload among people, but people lacked trusted sources. Policies to social distancing were disconnected from communities' abilities to follow such measures.
47	May 2020	Ghana	To assess the public knowledge, risk perception and preparedness to respond to the COVID-19 pandemic in the early stage of the outbreak in Ghana.	Quantitative online survey	People >18years (350)	More than half (62.7%) had good knowledge about the pandemic. Risk-perception was also high (68.3%). Regarding the preparedness to control and prevent COVID-19, 32.3% of the respondents regularly used a surgical mask, 91.4% washed their hands more than 3 times per day, 89.4% frequently used soap and water while washing their hands, 72.3% used sanitizers to disinfect their hands every time but only 6% regularly touched their faces.
48	May 2020	Kenya	To highlight some of the factors contributing to challenges faced by low-income countries in controlling the spread of this disease.	N/A	Article	There were rumours and misinformation; difficulties in implementing lockdowns; stigmatisation and social hostility; myths, perceptions and beliefs; lack of testing and inadequate medical facilities.
49	March 2020	Ethiopia	To assess the knowledge, perceptions and practices among the Jimma University Medical Center visitors in Jimma town.	Quantitative survey. Data were collected through face-to-face interviews.	Clients and patients (247)	41.3% of the respondents had high knowledge of COVID-19, defined as respondents scoring . 11 of 14 items covering issues such as symptoms, risk conditions, prognosis, modes of transmission and safety, and precautions in COVID-19. Majority (68.8%) felt able to control the disease. Frequent handwashing (77.3%) and avoidance of shaking hands (53.8%) were the dominant practices.
50	April 2020	12 African countries	To assess the perceptions of the COVID-19 pandemic in 12 African countries.	Quantitative survey. Data were collected through SMS and mobile web.	Literate adults with access to a mobile phone (4788)	Level of awareness about the disease was high (100%) at the start of the pandemic. Risk-perception was also high (72%), though varied across countries. The risk perception was lowest in Tanzania (50%) and highest in Mozambique (82%). Handwashing was the major preventive measure practiced, reported at 55% across the 12 countries.
51	August 2020	Nigeria	To perform an analysis of the COVID-19 pandemic in Nigeria and its prevention and control within the first two months of the outbreak	Review	N/A	Preventive efforts were undermined by poor compliance attitude, misconceptions and myths, and distrust for government. Some misconceptions reported were that the virus cannot thrive in Africa because of its hot climate; the disease is meant for the rich and the politicians and that it meant the coming of the Anti-Christ.
52	June 2020	Ethiopia	To assess the prevention knowledge and practices towards the COVID-19 among the residents of Ethiopia	Quantitative survey. Data were collected through Google forms, social media and email.	Ethiopian residents ≥18 (341)	Knowledge on COVID-19 prevention was high (91.8%). Social media was the main source of information. Prevention practices of the participants towards COVID-19 stood at 61% and 84% for social distancing and frequent handwashing, respectively.
53	June 2020	Ethiopia	To assess knowledge, attitudes and practice towards COVID-19 among patients with chronic disease	Quantitative survey. Data were collected through face-to-face interviews.	Chronic disease patients (404)	Knowledge and practices towards the disease were poor at 33.9% and 47.3%, respectively.
54	July 2020	Ethiopia	To investigate the knowledge, attitudes and practices toward COVID-19 following the introduction of state of emergency by the Ethiopian government to curb the spread of the disease	Quantitative phone survey	Citizens (1570)	Level of knowledge was reported to be inadequate, at 42%. Being a rural resident, older than 50 years, having at least primary education, were found to be the independent predictors of knowledge level. There was also observed poor adherence to recommended preventive measures, i.e., 31% of the respondents never washed their hands with soap, 81.4% never kept the 2-meter social distancing and 42.9%
55	June 2020	Malawi	To assess knowledge and behaviours related to the COVID- 19 pandemic in Malawi.	Quantitative phone survey	General population (630)	Misconceptions about mode of transmission of the disease, course and severity, were common. Some misconceptions reported included the virus being bloodborne (37%), others believed it was waterborne (55.2%), especially among the rural populations. Handwashing (>95%) and avoidance of crowds (50%) were the most reported preventive strategies.
56	April 2020	Nigeria	To determine the knowledge and practice of preventive measures against COVID-19 infection among pregnant women attending prenatal care in Abakaliki	Quantitative survey. Data were collected through face-to-face interviews.	Pregnant women (284)	60.9% of the respondents had adequate knowledge on preventive measures. Adequate knowledge was defined as participants who scored . 8 of 12 on the knowledge of coronavirus disease preventive measures questionnaire. However, the overall prevention practices were poor at 31.3%. Poor practices were defined as women who scored below 12 on the practice of coronavirus disease preventive measures questionnaire.
57	May 2020	Tanzania	To investigate KAP towards COVID-19 among residents in Tanzania during the April-May 2020 epidemic period.	Quantitative online survey	Tanzanian residents (400)	Majority, 84.4%, of participants had good knowledge, significantly associated with education level. Good knowledge was measured with a score above 8 of 12 of the total knowledge score. Prevention practices were avoidance of crowds (77%) and putting on face mask when going out (80%).
58	July 2020	Nigeria	To determine the levels of KAP toward COVID-19 among residents of north-central Nigeria	Quantitative online survey	Nigerian residents (589)	Respondents had good knowledge (99.5%) of COVID-19, gained mainly through social media and TV. Recommended practices of social-distancing, improved personal hygiene and use of face masks were good at 92.7%, 96.4% and 82.3%, respectively.
59	May 2020	Zimbabwe	To explore the prospects and challenges of traditional leaders in combating the COVID-19 novel virus in vulnerable rural communities in Zimbabwe.	Qualitative study	Grey and white literature review. Traditional leaders and public health officials (8)	Illiteracy of traditional leaders can be a major hindrance to uptake of COVID-19 prevention measures among the people that believe in them.
60	September 2020	Nigeria	To measure the level of COVID- 19 knowledge, attitudes and practices of the Nigerian public.	Quantitative online survey	Educated Nigerians with internet access (1,015)	Knowledge level of COVID-19 was good (78.7%); graded as 18–25 (>70%) of the total knowledge score of 25 points. Most respondents expressed positive attitudes (>90%). 22.5% of the respondents wore a face mask, and up to 90% washed their hands at least twice a day.
61	September 2020	Cameroon	To evaluate the factors influencing the knowledge, attitudes, and practices of Cameroonian respondents on COVID-19	Quantitative online survey	General population (1,006)	Social distancing (83.8%), use of face masks (100%) and handwashing (94.5%) were practiced as recommended.
62	September 2020	Nigeria	To examine COVID-19 related knowledge, attitude, and preventive practices among adult residents of Onitsha city, Nigeria.	Quantitative online survey	Adult residents (140)	60.7% of the respondents had high knowledge. High knowledge was defined as a score of ≥ mean score of 12.97 of 20 variables used to score total knowledge. Regular washing of hands was reported by 77.1% of the participants, and only 32.1% used face masks.
63	September 2020	Uganda	To assess the KAP and perceptions toward COVID-19 and face-mask use among high-risk individuals in Kampala, Uganda.	Quantitative survey. Data were collected through face-to-face interviews.	High-risk individuals, i.e., market food vendors, traffic officers, healthcare workers (644)	Nearly all (99.7%) participants reported having heard about COVID- 19. Age and receipt of information on face-mask use were significantly associated with knowledge on right procedure to wear a face-mask. With attitudes, majority (87.3%) agreed that face masks are a good protective measure against COVID-19. More than half (70.6%) wore face masks, and 81.4% practiced handwashing with soap and water.
64	May 2020	South Africa	To assess South Africans' understanding of and response to	Quantitative online survey	General population (55,823)	Knowledge of COVID-19 symptoms and incubation period was high (83.4% and 90%, respectively). Sources of information were government sources, TV and news websites. Risk-perception varied
64	May 2020	South Africa	To assess South Africans' understanding of and response to COVID-19 during the first week of the country's lockdown period.	Quantitative online survey	General population (55,823)	Knowledge of COVID-19 symptoms and incubation period was high (83.4% and 90%, respectively). Sources of information were government sources, TV and news websites. Risk-perception varied as low (38.8%), moderate (36.6%) and high at 24.6%, all of which varied by age, population group and dwelling type. The prevalence of high-risk perception was significantly lower in the youngest age group (18 – 29-year-olds).

#### Meta-analysis

**Figure 2:**
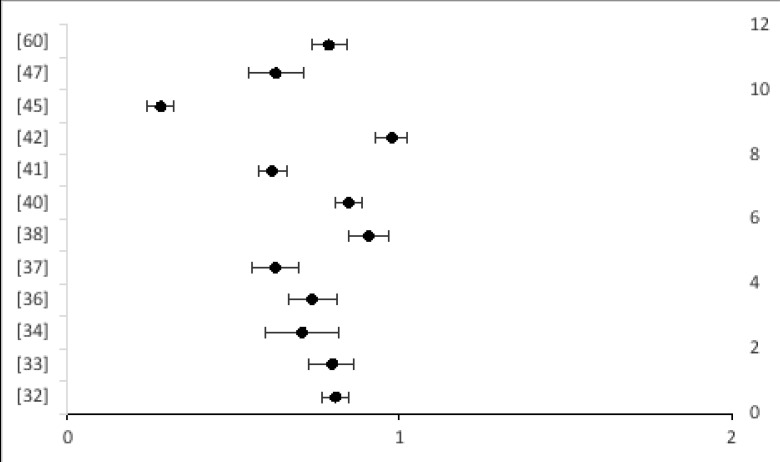
Forest plot of the studies that assessed knowledge at 95% confidence interval.

We ran a meta-analysis on the studies that assessed knowledge levels of participants on COVID-19. We calculated the weighted effect sizes of the studies, and determined the effect summary. We then determined heterogeneity of the studies, and assumed a fixed effects model. 95% Confidence Intervals of the studies were calculated, and a forest plot constructed (see Figure 2). The following equations^29^, ^30^ were employed in Microsoft Excel to guide the calculations:

**Effect size**, es = nevents/ntotal

**Weighted effect size** = w*es; where w = 1/(SE)2

**SE** = es/√es*n

**Q** = ∑(w*es2) − [[∑(w*es)]2/∑w]

**Effect summary**, e = ∑(w*es)/∑w

## Results

Figure 1 shows that 466 study titles were identified for this scoping review. On further screening, 140 study titles were found to be duplicates and were excluded. After screening the study abstracts, we excluded an additional 111 studies whose content was irrelevant to this scoping review. Furthermore, after conducting a full-text review of the remaining articles, we excluded 68 studies whose content was equally irrelevant to the review. Thirty-six (36) studies were considered for the final review.

### Characteristics of the studies reviewed

Table 1 presents an overview of the study characteristics. All the articles reviewed were published between March and October 2020 and all were published in the English language. Most studies (89%) reported on data collected between March and May 2020. A majority (92%) of the reviewed articles were studies. More than half (62%) of the reviewed studies were conducted through online links created using Google forms and sent to respondents through social media. More than three quarters (79%) of the reviewed studies were quantitative and employed a cross-sectional study design. The sample sizes of the studies reviewed ranged from 20 to 55,823 participants; with the lowest sample size being from a qualitative study conducted in Zimbabwe^31^. Almost a quarter (21%) of the studies were conducted in Nigeria. Most of the studies (62%) reported on knowledge, attitudes and practices with regard to COVID-19.

Table 2 presents the results from the meta-analysis of the studies that assessed knowledge. The average effect summary was 0.73 with a standard normal deviate of ±0.008, at (0.72–0.75) 95% Confidence Interval. The chi-square value, Q, was 8.45, indicating homogeneity of the studies.

**Table 2:** Meta-analysis for studies that assessed knowledge of COVID-19

Study	Events	Sample size	Outcome (es)^a^	Lower CI	Upper CI
[32]	1630	2009	0.81	0.770644212	0.849355788
[33]	578	722	0.8	0.734757219	0.865242781
[34]	161	227	0.71	0.600384412	0.819615588
[36]	403	546	0.74	0.667843475	0.812156525
[37]	350	559	0.63	0.564200826	0.695799174
[38]	901	987	0.91	0.85048613	0.96951387
[40]	1979	2336	0.85	0.812612269	0.887387731
[41]	885	1437	0.62	0.579287897	0.660712103
[42]	1721	1763	0.98	0.933789257	1.026210743
[45]	153	545	0.28	0.235574039	0.324425961
[47]	220	350	0.63	0.546844243	0.713155757
[60]	799	1015	0.79	0.735318981	0.844681019
Effect summary			0.729166667		

Standard error(fixed)	0.008				

C.I(fixed)^b^				0.72	0.75

a es, effect size

b C.I, confidence interval

### Narrative synthesis

Our review findings are presented in six a priori themes, including: a) Knowledge of COVID-19, b) COVID-19 risk-perception, c) COVID-19 misconceptions, d) COVID-19 conspiracy theories, e) Level of trust of COVID-19 information, and f) Uptake of COVID-19 prevention measures, as shown below.

#### a) Knowledge of COVID-19

**Figure 3:**
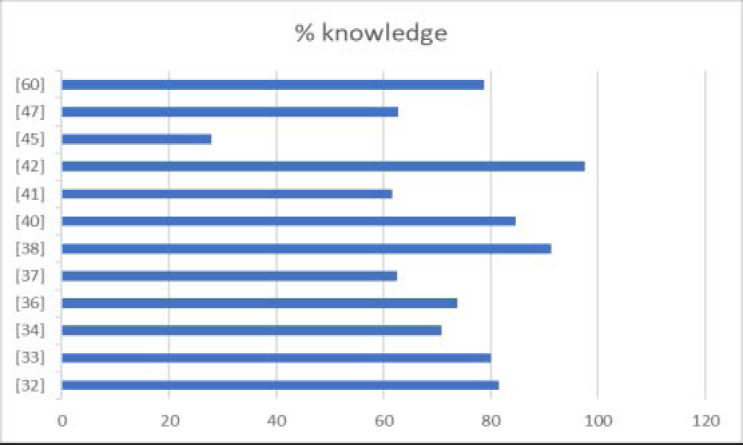
Bar graph showing percentage knowledge levels reported in the different studies.

Twelve (12) studies reported on knowledge of COVID-19^32^–^34^, ^36^, ^37^, ^38^, ^40^, ^41^, ^42^, ^45^, ^47^, ^60^. Figures 2 and 3 present a forest plot at 95% confidence interval, and bar graph of the studies that assessed knowledge generically. Across studies, we found that the majority (100%) of the study participants were aware of what COVID-19 is, what causes it and how it can be prevented. In two of the studies 32, 34, generic questions were asked about knowledge (e.g. what is the source of COVID-19? What are the transmission modes? What are the symptoms?); however, in the other ten studies 33, 36–38, 40, 41, 42, 45, 47, 60, there was an attempt to measure knowledge by using defined knowledge scores. It is important to note that the assessment of knowledge of COVID-19 was not standardized across studies.

Knowledge of COVID-19 ranged from 11.93% 45 to 100% 50. The main COVID-19 information sources were: social media; TV/radio; friends; workplace; newspapers; international organizations like the WHO and the US CDC; and the country's Ministry of Health 32–34, 36, 37, 45, 47,60. Only one study conducted in Kenya 32 assessed how much participants trusted the information source, and a majority (90%) of the participants trusted government messages on television, radio and text messages. Friends, family members, and acquaintances were least trusted. A few studies did not assess the source of information on COVID-19 from the study participants^38^, ^40^, ^42^, ^65^.

#### b) COVID-19 risk-perception

Evidence shows that risk-perception is a predictor of behaviour; that is, people will respond to recommended actions to avert a health threat depending on how they perceive the likelihood of facing the threat in question^14^. Thus, people's perception of the likelihood of contracting COVID-19 influences the way they respond to COVID-19 prevention measures. Studies measured risk-perception by asking questions about self-efficacy, collective efficacy, and stigma. Evidence relating to risk-perception was reported in most of the reviewed studies 12, 20, 31, 32, 36, 43, 46, 50, 51, 64, and it was found to vary from low to high (i.e. 35%–73.3%) 32, 45. Three of these studies, however, reported a low-risk perception, especially at the start of the pandemic in the respective countries^32^, ^51^, ^64^. Risk-perception was disaggregated based on gender, family, age and location 12, 45, 49, 50. Men felt that they were more vulnerable to COVID-19 compared to women 12. In contrast, Maredia 50 reported that the level of concern was higher among women than men. With age, the evidence shows that respondents who were younger (i.e. 18–29 year-olds) had a lower risk-perception^64^. A study by Aruhomukama et al. 63 reported that people aged 64 years and older, who are a high-risk group, had a poor perception of risk to COVID-19. Austrian and colleagues reported that respondents felt that they could not transmit the virus if they were infected 32. In Ethiopia, majority, 83.8% (n=207), perceived COVID-19 to be a disease leading to social stigma 49, and this study was similar to another study by Abdelhafiz et al. 37 who found that 22.7% of the respondents believe that the virus is associated with stigma. Perceived fear towards the virus also came up in some studies, with the major fear highlighted being death from COVID-19 42, 46.

#### c) COVID-19 misconceptions

Nine articles 12, 31, 35, 44, 46, 48, 51, 65, 66 reported about COVID-19 misconceptions. In Nigeria, for instance, people believed that they were immune to the virus due to Africa's hot climate, but also due to the fact that the disease was considered to affect rich people 46, 51. This same paper also reported on other misconceptions regarding prevention of COVID-19 such as steaming oneself with hot water, drinking of gins, gargling the throat with salty water, consumption of hot drinks made with ginger, pepper, lemon, garlic and all manner of herbs perceived to prevent the disease. An article that appeared on the BBC website 35 reported similar findings: that steaming & alcohol consumption were used as a cure of COVID-19; but also that some African communities believed that the disposable blue facemasks were contaminated with the virus. Tangwa et al. 44 highlighted the introduction of supposed cures of COVID-19, i.e., the COVID Organics cure, whose use warranted tests of efficacy. Use of chloroquine as a proposed cure to COVID-19 despite the undefined efficacy and safety of the drug was also reported in two papers 51,66. This notwithstanding, evidence by Soumare and Darras^67^ suggested that chloroquine had been used in combination with other drugs in the treatment of COVID-19 patients in Cameroon, Senegal, Burkina Faso, Algeria and Morocco. Use of prayers was another misconception that came up from one of the reviewed articles, where there was promotion of prayers for divine intervention against the virus 12.

#### d) Conspiracy theories about COVID-19 in SSA

Mixed beliefs about the origin of COVID-19 were noted among SSA populations. Notably, Adenubi et al. 34 reported that African populations believed that COVID-19 was a biological weapon created in China 35. This is in consonance with studies conducted in Nigeria and Egypt^37^, ^46^. In Nigeria, Isah et al. 33 reported that 36% of the respondents believed that COVID-19 was created in a laboratory, aimed at depopulating the world. Olapegba and colleagues further report that respondents in Nigeria thought that COVID-19 is a plague caused by sins and misbelief of humans 46. The virus was also linked to 5G, a new world order; punishment from God and the coming of the Anti-Christ, but also that it was intended to kill Africans 51. Surprisingly, this information was reportedly being fuelled by highly respected and educated people 51. This finding was similar to an article published in a London School of Economics blog where people believed that the virus is a curse from God 68. Although these conspiracy theories seemed to be common in most countries where the reviewed studies were conducted, studies done by Kebede et al. 49, Aynalem et al. 36, Elnadi et al. 41 and Maredia 50 show that a majority of the population had correct information about the disease. For instance, Kebede and colleagues found that about 95.1% of the population knew that the COVID-19 virus spreads via respiratory droplets of infected people while Elnadi et al. 41 found that 68.9% had a positive attitude towards protective measures recommended by the WHO or their local health authorities.

#### e) Level of trust of COVID-19 information

Issues relating to trust were reported in four studies 31,43, 51, 64, and these were about trust in the capacity of the healthcare system to adequately manage complex cases of COVID-19. Furthermore, respondents alluded to the fact that COVID-19 was a political case aimed at attracting international funding. In Uganda, anecdotal evidence suggests that people believe that the surging number of cases of the virus is falsified, and is a political move by the government to seek additional funding 69. A recent investigative report published in Uganda's daily newspaper, the Daily Monitor, revealed that deaths due to COVID-19 were from a faulty oxygen machine as well as other loopholes in the country's health system 69. Confidence in media coverage to provide adequate and correct information also came up in another study conducted in Egypt 37. The reporting in these studies showed that because the populations did not fully trust both the government and the healthcare system to manage COVID-19, their risk-perception towards the disease was low.

#### f) Uptake of COVID-19 prevention measures

Eleven (11) studies 31, 52–59, 61, 63 reported about uptake of COVID-19 prevention measures. The overall uptake of recommended COVID-19 prevention measures was reported to be poor by three studies 52, 54, 56, as majority (43%–77.4%) of the respondents never practiced COVID-19 prevention measures. Nevertheless, when we examined uptake of individual COVID-19 prevention measures, we found varying levels of uptake, as shown below.

### Hand-washing using water and soap / hand sanitizing

Seven (7) of 11 studies 52–56, 61, 62 reported on hand washing/hand sanitizing. The majority (6 of 7) of the studies^52^–^55^, ^61^, ^62^ with content on washing hands using water and soap/hand sanitizing reported that more than 95% of the respondents washed their hands using water and soap /hand sanitizers. Only one (1) of the seven (7) studies 56 reported that less than a third (26.8%) of the respondents washed their hands using water and soap / hand sanitizers.

### Use of face mask

The practice of wearing a face mask was reported by six (6) of 11 studies 52, 53, 55, 56, 61, 62. Although a study by 61 reported that all (100%) of the participants wore a face mask, the majority (5) of the six studies 52, 53, 55, 56, 62 reported that less than half (24%–36.6%) of the participants wore a face mask when leaving their homes. Furthermore, a study conducted in Malawi 55 reported a higher facemask use among urban residents (22.5%) as compared to rural residents (5%).

### Maintaining social distancing

Most studies included questions on social distancing or avoiding crowded places. Seven (7) studies 41, 31, 36, 46, 52,53, 55 reported on social distancing alone; four (4) studies^45^, ^54^, ^55^, ^58^ reported on avoiding crowded places alone, while one study 54 reported about both social distancing and avoiding crowded places. Studies that reported on social distancing alone found that the level of social distancing varied from 20.4% to 83.8% among respondents. Studies that reported on avoiding crowded places alone found that there was a high uptake of this measure among respondents (50%–77%) while studies that reported on both social distancing and avoiding crowded places found that more respondents (48.4%) avoided crowded places as compared to those that practiced social distancing (16.8%). Three studies 53, 54, 56 reported that less than a third (18.6%–29.9%) of the respondents maintained the recommended 1-meter distance between themselves and other individuals. The highest social distancing practice was reported in a study by Elnadi et al. 41, and it was at 96% among the respondents. The practice of avoiding crowded places was reported by four (4) studies; two (2) studies 57, 58 reported that about three quarters (72.5% and 77%) of the respondents avoided crowded places; one (1) study 55 reported that half (50%) of the respondents avoided crowded places while another study 54 reported that less than half (48.4%) of the respondents avoided crowded places. A qualitative study 76 conducted in Zimbabwe reported inadequate practice of social distancing as residents queue body-to-body to access communal water and the staple food.

### Other adopted ways of preventing COVID-19

Other than the three COVID-19 prevention measures referred to above, some studies reported other practices that were undertaken by respondents to prevent COVID-19. For example, Nwafor et al. 56 reported that 21.5% of the respondents avoided touching their eyes, nose and mouth with their hands while 17.3% stayed indoors. In another study, almost half (45.3%) of the participants believed in prayer as a COVID-19 prevention measure 58. Akalu et al. 53 found that 72% of the respondents weren't shaking hands to avoid contracting COVID-19 while more than half (55.2%) cleaned and disinfected frequently touched objects and surfaces. Cowen 59 reported that some people opted to migrate from urban areas to rural areas in order to reduce chances of COVID-19 infection. Iloanusi et al. 62 reported that 44.3% of the respondents avoided touching the face, 42.1% cleaned surfaces regularly while 40% cancelled travel plans. Ngwewondo et al. 61 reported that 20% of the respondents were confined at home, 74.6% ate citrus fruits and took vitamin C tablets, 35.9% resorted to traditional concoctions, while some participants decided to self-medicate themselves with chloroquine (4.4%) and paracetamol (4.6%).

## Discussion

Our scoping review of COVID-19 knowledge, risk-perception, misconceptions, conspiracy theories and uptake of COVID-19 prevention measures in SSA shows that: a) knowledge of COVID-19 as a disease was high (ranging between 91.3–100%); b) COVID-19 risk-perception was high (ranging from 73.3% - 86.9%) but misconceptions and conspiracy theories about COVID-19 abound; c) uptake of all the three primary COVID-19 prevention is generally poor (2.7% - 55%) across studies, although uptake of hand-washing with soap and water is relatively high compared to wearing of face-masks and social distancing or avoiding crowded places. Collectively, these findings suggest a need for enhanced health promotion to improve uptake of COVID-19 recommended measures if people in SSA are to reduce the risk of getting infected with COVID-19.

Our finding that knowledge of COVID-19 as a disease is high among people in SSA could be attributed to the numerous efforts by respective governments and relevant stakeholders in educating masses about the disease across several platforms such as television, radio stations and social media 32, 37, 70. Also, at the time of conducting these studies, all of these SSA countries were in the early stages of the outbreak and implemented strategies based on experiences from the Western world. However, the evidence base relating to knowledge of transmission mode, signs and symptoms and preventive measures was very small, as majority of the studies never went further to assess these variables. These studies never established the knowledge levels and the proportion of participants with either good or poor knowledge. This leaves an unfilled information gap in the evidence base that would be important in informing programming and development of context-specific prevention and control strategies. Another review however argues that although high-level knowledge about a disease is reported, it rarely leads to an increase in uptake of preventive services, especially when the perception of risk to that disease is low 14. Nevertheless, our findings show that knowledge of COVID-19 is universal, suggesting that people in SSA know how the corona virus is transmitted and how it can be prevented. We found that risk-perception towards COVID-19 was high (i.e., 73.3–86.9%) despite the severe effects linked to the infection. Many factors influence risk-perception; social, cultural or psychological factors and these equally have an influence on people's access to factual information about a disease, their perceived susceptibility and severity or their prevention practices 14. In this review, the high risk-perception could be because many of these studies were carried out at a time when the disease etiology was still new to many, and most SSA countries were within the containment phase of the epidemic curve 71, having learnt lessons from Asia and Europe. Notably, studies where risk-perception was recorded as high were conducted between March and May 2020. In contrast, where risk-perception was low, we could attribute it to the fact that the outbreak was unfolding in a tense socio-economic context, which resulted in the distrust of some of the messages from public authorities. Consequently, the rules of public hygiene and containment were not followed 72. The other reason that could explain the observed low risk-perception as reported by some studies was the promulgation of misconceptions and conspiracy theories about the disease, amidst the many political and health system capacity challenges to effectively handle the pandemic.

Overall, the uptake of recommended COVID-19 prevention measures was poor. For instance, the practice of social distancing, hand washing with soap and water/hand-sanitizing and wearing a face mask only ranged between 2.7–43%. A recent study among 2,500 adolescent boys and young men in Kampala, Uganda, found that although knowledge of at least two COVID-19 prevention measures was high (>80%), only 22.2% reported that they always wore a face mask while in a public place; 40.9% always washed their hands with soap and running water while 17.6% always avoided gatherings of more than five people^79^. In many African contexts, it is almost impossible to observe social distancing. Informal settlements and central business regions with crowded populations pose a problem for people accessing these areas and therefore, the concept of social distancing sounds almost unfeasible^73^. This finding could as well be because some of the reviewed studies were conducted in the months after mid-April 2020 when the level of risk-perception and fear of COVID-19 had started to reduce 51, 64. The poor uptake of the recommended COVID-19 prevention measures is of great public health concern and may increase the incidence rate of COVID-19 infections in SSA^1^. This may later overwhelm the existing fragile healthcare systems 74, thus leading to a hike in the COVID-19 fatalities within the region.

Interestingly, uptake of hand-washing with soap and water/hand sanitizing was relatively higher than the uptake of other COVID-19 prevention measures. A majority of the studies reported that most of the respondents washed their hands using soap and water or sanitized their hands. However, while this practice was high, it was mainly observed in the first few months of the pandemic^32^, ^49^. Presently, uptake of COVID-19 prevention measures has been abandoned in some settings and populations. The complacency is now visible everywhere, making it an avenue for contact transmission of the virus^75^. Similarly, while hand washing is easily applicable to middle- and high-income countries, the same cannot be assumed for the African context where water access is still a challenge^76^, ^77^. The same is true with the wearing of face masks whose use was also found to be low^78^.

### Implications for Research and Policy

Given the low vaccine coverage in sub-Saharan Africa, with only 7.4% of the African population fully vaccinated, there is a need for research on alternative ways to improve uptake of non-pharmaceutical interventions whose uptake remains equally low across countries and populations. In addition, other non-pharmaceutical interventions such as stay-at-home requirements, international travel controls, contact tracing as well as income support are still necessary to reduce transmission^80^, ^81^. Currently, we observe an increase in vaccine hesitancy among populations, and with the unease in the uptake of non-pharmaceutical interventions, it is likely that countries will experience severe cases of COVID-19 again. Therefore, mass vaccination should be made mandatory, and integrated with the non-pharmaceutical interventions that play an important role in virus containment 82. This scoping review has revealed a dearth of quantitative studies measuring associations between factors that could be responsible for the observed practices towards COVID-19 prevention among populations in Sub-Saharan Africa. This implies that there is a missed opportunity for designing targeted interventions to curb the spread of the virus. Adhering to the primary prevention measures is critical in reducing contact transmission and severity due to COVID-19^81^, calling for targeted interventions to promote behavior change. Collectively, study findings on misperceptions and conspiracy theories imply that without efforts to overturn the infodemic, risk perception to future disease outbreaks will remain low. As such, findings call for a need to understand why such infodemic abounds, but also point to a need for social and behavioral change approaches necessary to enhance uptake of non-pharmaceutical interventions across countries and populations in SSA.

### Study Limitations and Strengths

Our study had some limitations and strengths. While we endeavored to search for published literature on this subject, and used internet sources for unpublished literature, there were few papers published on this subject from SSA. Our conclusions may thus not reflect the full breadth and width of the knowledge, risk perceptions, conspiracy theories and uptake of COVID-19 prevention measures across SSA. In addition, we observed that most of the papers used in this review pertained to data that were collected early into the epidemic (about March, April and May 2020); thus, the findings may not necessarily reflect the current COVID-19 prevention measures. It is likely that people's perceptions of COVID-19 could have changed with time, suggesting that our findings should be interpreted with caution. However, we believe that in most of SSA, people have continued to perceive the disease as non-existent and a disease of “other people”, suggesting that our findings might reflect the current thinking and adoption of COVID-19 prevention practices. Despite the above-mentioned limitations, our study contributes to existing literature on COVID-19 by identifying key themes arising out of knowledge, risk-perception and uptake of recommended preventive measures in SSA. This information is essential in informing COVID-19 prevention measures in SSA. Our review also identified potential gaps that could be addressed in further research.

## Conclusion

Our review shows that knowledge and risk-perception were high, especially at the start of the pandemic, but uptake of preventive measures was generally low. Further research is warranted to improve the evidence base which is crucial for guiding strategic policy. Due to the continuous spread of misinformation, respective governments must continuously monitor information sources to ensure that correct and reliable information is disseminated to the public. Finally, we recommend more sensitization programs aimed at curbing complacency towards practicing preventive measures which, in itself, would become a major risk.

## References

[1] WHO (2020). Corona virus.

[2] Ministry of Health, Uganda (MOH) (2020). National Guidelines for Management of COVID-19.

[3] Perlman S (2020). Another Decade, another Corona virus. N Engl J Med.

[4] Shabu S, Amen KM, Mahmood KI, Shabila NP (2020). Risk perception and behavioral response to COVID-19 in Iraqi Kurdistan Region. Research Square.

[5] Koryo Tours List of countries without corona virus.

[6] Worldometer (2021). Corona virus live updates. Reported Cases and Deaths by Country, Territory, or Conveyance.

[7] British Broadcasting Corporation Covid map: Corona virus cases, deaths, vaccinations by country.

[8] Africa CDC Africa CDC COVID-19 Dashboard Available at: https://africacdc.org/covid-19/.

[9] World Health Organisation (WHO) (2020). Corona virus disease (COVID-19) pandemic.

[10] World Health Organisation (WHO) (2020). COVID-19 Strategic Preparedness and Response Plan in Operational Planning Guidelines to Support Country Preparedness and Response.

[11] Lal A, Erondu NA, Heymann DL, Gitahi G, Yates R (2020). Fragmented health systems in COVID-19: rectifying the misalignment between global health security and universal health coverage. The Lancet.

[12] Kasozi KI, Macleod E, Sempijja F, Mahero MW, Matama K, Musoke GH (2020). Misconceptions on COVID-19 Risk Among Ugandan Men: Results From a Rapid Exploratory Survey. Frontiers in Public Health.

[13] Hager E, Odetokun IA, Bolarinwa O, Zainab A, Okechukwu O, Al-Mustapha AI (2020). Knowledge, attitude, and perceptions towards the 2019 Corona virus Pandemic: A bi-national survey in Africa. PLoS One.

[14] Aduh U, Folayan M, Onyeaghala A, Ajayi I, Coker M, Tebeje Y (2020). Risk perception, public health interventions, and Covid-19 pandemic control in sub-Saharan Africa. Journal of Public Health in Africa.

[15] Sengeh P, Jalloh M, Webber N, Ngobeh I, Samba T, Thomas H (2020). Community knowledge, perceptions and practices around COVID-19 in Sierra Leone: a nationwide, cross-sectional survey. BMJ Open.

[16] Abdelhamid MT (2020). COVID-19 modeling tools will ‘help to fill knowledge gaps’.

[17] Ezeh A, Fonn S (2020). Sub-Saharan Africa needs to plug local knowledge gap to up its anti-COVID-19 game.

[18] Osuchowski MF, Aletti F, Cavaillon JM, Flohé S, Giamarellos-Bourboulis E, Huber-Lang M (2020). SARS-CoV-2/COVID-19: Evolving Reality, Global Response, Knowledge Gaps, and Opportunities. Shock. (Augusta, Ga.).

[19] British Broadcasting Corporation (BBC) Corona virus: Africa could be next epicentre, WHO warns, in Corona virus pandemic.

[20] Kaulu B, Kabala E, Mapoma R, Munyonzwe C (2020). Risk Perception, Behavioral Response to COVID-19, and the Mediating Role of Information Sources in Zambia. Southern African Journal of Policy and Development.

[21] Kirby B, Taru J, Chimbidzikai T (2020). Pentecostals are in a “spiritual war” against corona virus in Africa - as are some political leaders.

[22] Muhumuza R (2020). As Africa's COVID-19 cases rise, faith is put to the test.

[23] Manguvo A, Mafuvadze B (2015). The impact of traditional and religious practices on the spread of Ebola in West Africa: time for a strategic shift. The Pan African Medical Journal.

[24] Bangura JB (2020). Hope in the midst of death: Charismatic spirituality, healing evangelists and the Ebola crisis in Sierra Leone. Missionalia.

[25] Roura M, Nsigaye R, Nhandi B (2010). “Driving the devil away”: qualitative insights into miraculous cures for AIDS in a rural Tanzanian ward. BMC Public Health.

[26] Arksey H, O'Malley L (2005). Scoping studies: towards a methodological framework. International Journal of Social Research Methodology.

[27] Tricco AC, Lillie E, Zarin W, O'Brien KK, Colquhoun H, Levac D (2018). PRISMA Extension for Scoping Reviews (PRISMA-ScR): Checklist and Explanation. Annals of Internal Medicine.

[28] Peters MDJ, Godfrey CM, Khalil H, Mclnerney P, Parker D, Soares CB (2015). Guidance for conducting systematic scoping reviews. International Journal of Evidence-Based Healthcare.

[29] Neyeloff J L, Fuchs S C, Moreira L B (2012). Meta-analyses and Forest plots using a Microsoft Excel spreadsheet: step-by-step guide focusing on descriptive data analysis. BMC Research Notes.

[30] Mikolajewicz N, Komarova S V (2019). Meta-Analytic Methodology for Basic Research: A Practical Guide. Frontiers in Physiology.

[31] Mackworth-Young C, Chingono R, Mavodza C, McHugh G, Chikwari CD, Weiss H (2020). ‘Here, we cannot practice what is preached’: early qualitative learning from community perspectives on Zimbabwe's response to COVID-19. Bulletin of the World Health Organization.

[32] Austrian K, Pinchoff J, Tidwell J, White C, Abuya T, Kangwana B (2020). COVID-19 related knowledge, attitudes, practices and needs of households in informal settlements in Nairobi, Kenya. World Health Organisation Bulletin.

[33] Isah MB, Abdulsalam M, Bello A, Ibrahim MI, Usman A, Nasir A (2020). Corona Virus Disease 2019 (COVID-19): Knowledge, attitudes, practices (KAP) and misconceptions in the general population of Katsina State, Nigeria. medRxiv.

[34] Adenubi OT, Adebowale OO, Oloye AA, Bankole NO, Ayo-Ajayi PO, Akinloye AK (2020). University Community-Based Survey on the Knowledge, Attitude and Perception about COVID-19 Pandemic: The Federal University of Agriculture, Abeokuta, Nigeria as a Case Study. Preprints.

[35] British Broadcasting Corporation (BBC) Corona virus: What false information has spread in Africa. BBC News.

[36] Aynalem YA, Akalu TY, Gebressellasie B, Sharew NT, Shiferaw WS (2020). Assessment of undergraduate student knowledge, practices, and attitude towards COVID-19 in Debre Berhan University, Ethiopia. Research Square.

[37] Abdelhafiz AS, Mohammed Z, Ibrahim ME, Ziady HH, Alorabi M, Ayyad M, Sultan EA (2020). Knowledge, Perceptions, and Attitude of Egyptians Towards the Novel Coronavirus Disease (COVID-19). Journal of Community Health.

[38] Mohamed A, Elhasan E, Mohamed A, Mohammed AA, Mahgoop MA, Sharif ME (2020). Knowledge, attitude and practice of the Sudanese people towards COVID-19: An online survey. BMC Public Health.

[39] Nicholas T, Mandaah FV, Esemu NS, Amana BTV, Kouam TDG, Lambou FV (2020). COVID-19 knowledge, attitudes and practices in a conflict affected area of the South West Region of Cameroon. Pan African Medical Journal.

[40] Mousa KNAA, Saad MMY, Abdelghafor MTB (2020). Knowledge, attitudes, and practices surrounding COVID-19 among Sudan citizens during the pandemic: an online cross-sectional study. Sudan Journal of Medical Sciences (SJMS).

[41] Elnadi H, Odetokun IA, Bolarinwa O, Zainab A, Okechukwu O, Al-Mustapha AI (2020). Knowledge, attitude, and perceptions towards the 2019 Coronavirus Pandemic: A bi-national survey in Africa. PLoS One.

[42] Ssebuufu R, Sikakulya F, Binezero S, Wasingya L, Nganza S, Bwaga I (2020). Awareness, knowledge, attitude and practice towards measures for prevention of the spread of COVID-19 in the Ugandans: A nationwide online cross-sectional Survey. MedRxiv.

[43] Lowe M (2020). Using rapid online survey to assess public perceptions of Covid-19 in Gambia. Pan African Medical Journal.

[44] Tangwa GB, Munung NS (2020). COVID-19: Africa's relation with epidemics and some imperative ethics considerations of the moment. Research Ethics.

[45] Bokagne A, Gilchrist KD (2020). COVID-19 knowledge, attitudes and practices in a conflict affected area of the South West Region of Cameroon. Pan African Medical Journal.

[46] Olapegba PO, Ayandele O, Kolawole S, Oguntayo R, Gandi J, Dangiwa A (2020). A Preliminary Assessment of Novel Coronavirus (COVID-19) Knowledge and Perceptions in Nigeria. medRxiv.

[47] Serwaa D, Lamptey E, Baffour A, Appiah EK, Senkyire J, Ameyaw K (2020). Knowledge, risk perception and preparedness towards coronavirus disease-2019 outbreak among Ghanaians: a quick online cross-sectional survey. Pan African Medical Journal.

[48] Akwa E (2020). Challenges to the Effective Prevention and Control of Covid-19 in Low Income Countries. OSF Preprints.

[49] Kebede Y, Yitayi Y, Birhanu Z, Mekonen S, Ambelu A (2020). Knowledge, perceptions and preventive practices towards COVID-19 early in the outbreak among Jimma university medical center visitors, Southwest Ethiopia. PLoS One.

[50] Maredia M (2020). Awareness, Risk Perceptions and Safety Behavior: How are Men and Women in Rural and Urban Africa Responding to COVID-19?.

[51] Omaka-Amari LN, Aleke CO, Obande-Ogbuinya NE, Ngwakwe PC, Nwankwo O, Afoke EN (2020). Coronavirus (COVID-19) Pandemic in Nigeria: Preventive and Control Challenges within the First Two Months of Outbreak. African Journal of Reproductive Health.

[52] Bekele D, Tolosa T, Gayesa R, Teshom W (2020). The knowledge and practice towards COVID-19 pandemic prevention among residents of Ethiopia: An Online Cross-Sectional Study. PLoS One.

[53] Akalu Y, Ayelign B, Molla MD (2020). Knowledge, Attitude and Practice Towards COVID-19 Among Chronic Disease Patients at Addis Zemen Hospital, Northwest Ethiopia. Infection and Drug Resistance.

[54] Negera E, Demissie TM, Tafess K (2020). Inadequate level of knowledge, mixed outlook and poor adherence to COVID-19 prevention guideline among Ethiopians. bioRxiv.

[55] Banda J, Dube A, Brumfield S, Amoah A, Crampin A, Reniers G (2020). Knowledge and Behaviors Related to the COVID-19 pandemic in Malawi. medRxiv.

[56] Nwafor JI, Aniukwu JK, Anozie BO, Ikeotuonye AC, Okedo-Alex IN (2020). Knowledge and practice of preventive measures against COVID-19 infection among pregnant women in a low-resource African setting. International Journal of Obstetrics and Gynaecology.

[57] Rugarabamu S, Byanaku A, Ibrahim M (2020). Knowledge, attitudes, and practices (KAP) towards COVID-19: A quick online cross-sectional survey among Tanzanian residents. medRxiv.

[58] Reuben RC, Danladi MMA, Saleh AD, Ejembi EP (2020). Knowledge, Attitudes and Practices Towards COVID-19: An Epidemiological Survey in North-Central Nigeria. Journal of Community Health.

[59] Cowen D (2020). Prospects and Challenges for Traditional Leaders in Combating COVID-19 Pandemic in Rural Zimbabwe.

[60] Adesegun OA, Binuyo T, Adeyemi O, Ehioghae O, Rabor DF, Amusan O (2020). The COVID-19 Crisis in Sub-Saharan Africa: Knowledge, Attitudes, and Practices of the Nigerian Public. The American Journal of Tropical Medicine and Hygiene.

[61] Ngwewondo A, Nkengazong L, Ambe AL, Ebogo JT, Mba FM, Goni HO (2020). Knowledge, attitudes, practices of/towards COVID 19 preventive measures and symptoms: A cross-sectional study during the exponential rise of the outbreak in Cameroon. PLoS Neglected Tropical Diseases.

[62] Iloanusi NR, Iloanusi S, Mgbere O, Ajayi A, Essien EJ (2020). COVID-19 Related Knowledge, Attitude and Practices in a Southeastern City in Nigeria: A Cross-Sectional Survey.

[63] Aruhomukama D, Musoke D, Mboowa G, Bulafu D (2020). Face-masking, an acceptable protective measure against COVID-19: Findings of Ugandan high-risk groups. American Journal of Tropical Medicine & Hygiene.

[64] Reddy SP, Sewpaul R, Mabaso M, Parker S, Naidoo I, Jooste S (2020). South Africans' understanding of and response to the COVID-19 outbreak: An online survey. South African Medical Journal.

[65] Ibrahim O, Ekundayo D (2020). COVID-19 Pandemic in Nigeria: misconception among individuals, impact on animal and the role of mathematical epidemiologists. Preprints.

[66] Abena PM, Decloedt EH, Bottieau E, Suleman F, Adejumo P, Sam-Agudu NA (2020). Perspective Piece Chloroquine and Hydroxychloroquine for the Prevention or Treatment of Novel Coronavirus Disease (COVID-19) in Africa: Caution for Inappropriate Off-Label Use in Healthcare Settings. American Journal of Tropical Medicine and Hygiene.

[67] Soumaré M, Darras R (2020). Corona virus: Didier Raoult the African and chloroquine, from Dakar to Brazzaville.

[68] Storer L, Osuta J, Angualia D (2020). Do COVID-19 conspiracy theories challenge public health delivery.

[69] Kalinaki D (2020). The Daily Monitor: INVESTIGATION: How lack of oxygen, poor health sector is killing COVID-19 patients in Uganda.

[70] Olum R, Chekwech G, Wekha G, Nassozi DR, Bongomin F (2020). Coronavirus Disease-2019: Knowledge, Attitude, and Practices of Health Care Workers at Makerere University Teaching Hospitals, Uganda. Frontiers of Public Health.

[71] Mburu Y, Boum Y (2020). Coronavirus: Amid the global pandemic, lessons for Africa. Brookings News.

[72] Delamou A, Sidibe S, Camara A, Traore MS, Toure A, Van-Damme W (2020). Tackling the COVID-19 pandemic in West Africa: Have we learned from Ebola in Guinea?. Preventive Medicine Reports.

[73] Noko K (2020). In Africa, social distancing is a privilege few can afford.

[74] Velavan T P, Meyer C G (2020). The COVID-19 epidemic. Tropical Medicine & International Health.

[75] Mutsaka F (2020). Progress against virus brings complacency in parts of Africa. The Washington Post: Associated Press.

[76] Torti C, Mazitelli M, Trecarichi EM, Owachi D (2020). Potential implications of SARS-CoV-2 epidemic in Africa: where are we going from now?. BMC Infectious Diseases.

[77] Olu OO, Waya JLL, Maleghemi S, Rumunu J, Ameh D, Wamala JF (2020). Moving from rhetoric to action: how Africa can use scientific evidence to halt the COVID-19 pandemic. Infectious Diseases of Poverty.

[78] Aloui-Zarrouk Z, El-Youssfi L, Badu K, Fagbamigbe AF, Matoke-Muhia D, Ngugi C (2020). The wearing of face masks in African countries under the COVID-19 crisis: luxury or necessity?. AAS Open Research.

[79] Matovu JKB, Kabwama SN, Ssekamatte T, Senkusu J, Wanyenze R (2021). COVID-19 Awareness, Adoption of COVID-19 Preventive Measures, and Effects of COVID-19 Lockdown Among Adolescent Boys and Young Men in Kampala, Uganda. Journal of Community Health.

[80] Liu Y (2021). The impact of non-pharmaceutical interventions on SARS-CoV-2 transmission across 130 countries and territories. BMC Medicine.

[81] Olaf M, Oliver R, Albrecht J (2021). Effects of non-pharmaceutical interventions against COVID-19 on the incidence of other diseases. The Lancet.

[82] Gozzi N, Bajardi P, Perra N (2021). The importance of non-pharmaceutical interventions during the COVID-19 vaccine rollout. medRxiv.

